# Legacy Effects of Different Preceding Crops on Grain Yield, Protein Fractions and Soil Nutrients in Subsequent Winter Wheat

**DOI:** 10.3390/plants14162598

**Published:** 2025-08-21

**Authors:** Rui Wang, Jiayun Wu, Yang Wang, Zhimei Sun, Wenqi Ma, Cheng Xue, Huasen Xu

**Affiliations:** 1 College of Resources and Environment Science (College of Land Resources), Hebei Agricultural University, Baoding 071001, China; wangrui220811@163.com (R.W.); 13013247510@163.com (J.W.); xiaoyiranwy85@163.com (Y.W.); sunzhm2002@163.com (Z.S.); mawq@hebau.edu.cn (W.M.); xuecheng@hebau.edu.cn (C.X.); 2 Key Laboratory for Farmland Eco-Environment of Hebei, Hebei Agricultural University, Baoding 071001, China

**Keywords:** wheat, yield, protein, soil fertility, preceding crop effect, synergistic benefit

## Abstract

Given the pressing global food security crisis and climate change-induced constraints on agricultural productivity, crop rotation proves critical for boosting yield and grain quality of winter wheat (*Triticum aestivum*) alongside ameliorating soil quality. However, the legacy effect of different preceding crops on synergistic increments of wheat productivity and soil fertility remains to be fully clarified. Five different preceding crop–winter wheat rotations were conducted in a field experiment established in Huanghua, China. Maize (*Zea mays*), sorghum (*Sorghum bicolor*), and millet *(Setaria italica*) were designated as preceding gramineous crops, and soybean (*Glycine max*) and mung bean (*Vigna radiata*) were assigned as preceding legume crops. Grain yield, protein fraction, and soil nutrients were measured to elucidate the legacy effect of the preceding crops on the subsequent winter wheat. Leguminous predecessors significantly evaluated the grain yield of winter wheat compared to gramineous predecessors, particularly that the mung–winter wheat rotation (Mun-W) was 11.56% higher than that of the maize–winter wheat rotation (Mai-W). This rising yield was attributed to the increase of 4.05% in spike number per hectare and 14.31% in kernel number per spike. The Mun-W facilitated the highest gluten protein content (8.22%) in winter wheat among five treatments, which was 6.06% higher than that in the sorghum–winter wheat system. Soil organic matter (SOM) showed an advantage in legume–winter wheat rotations (Leg-Ws) compared to gramineous crop–winter wheat systems (Gra-Ws). Notably among these, the Mun-W significantly enhanced SOM content by 0.99% relative to the Mai-W. The soybean–winter wheat system decreased soil pH by 0.36 compared to the Mai-W system. Coupling coordination degree (CCD) and co-benefit index (CBI) in the Leg-Ws exhibited significant superiority of 62.41% and 42.22% over the Gra-Ws, respectively, and the Mun-W attained maximum CCD by 0.84 and CBI by 0.77. From a multi-objective assessment perspective of the legacy effect of the preceding crops, legume-based rotations facilitate synergistic improvements of yield, protein quality, and soil nutrients in winter wheat.

## 1. Introduction

Wheat, as one of the most extensively cultivated staple crops worldwide, supplies 20% of caloric and protein intake for humanity and serves as a critical pillar of worldwide food security [1]. Projections indicate an 11% rise in global wheat demand by 2031 [2]. Recent data shows that concurrent droughts would cause wheat yield reductions [3]. Elevated CO_2_ concentrations coupled with thermal stress will degrade wheat grain quality, posing substantial threats to human nutrition security [4,5]. Crop rotation, a traditional yet practical measure for enhancing agroecosystem services, utilizes prior crops to stabilize and increase succeeding crop productivity and quality via improved soil structure, augmented fertility, and control of soil-borne pathogens [6,7]. Comprehensive meta-analysis has demonstrated that legume rotations significantly boost follow-on crop yield [8] and augment soil organic carbon content [9]. Consequently, investigating novel rotation approaches involving winter wheat holds significant importance for combating global food insecurity.

Nevertheless, such yield responses demonstrate marked heterogeneity across different pre-crop types. Leguminous crops enhance soil nitrogen levels through biological N fixation and high-quality rhizodeposition, while strategic root system complementarity between sequential crops improves water-nutrient use efficiency, N uptake, yield, and soil fertility in subsequent crops [10,11]. Peanut–wheat and soybean–wheat rotations boosted wheat equivalent annual yield, compared to unfertilized conventional maize–wheat rotation [12]. Succession wheat after millet residues showed significantly lower productivity than following pea crops [13]. Additionally, variations in pre-crop species induce marked heterogeneity in how yield constituents determine ultimate output. Legume–rapeseed rotations outperformed rice–rapeseed systems in seed yield, while exhibiting significantly enhanced root collar thickness, plant stature, primary fertile branch height, and productive branch count at physiological maturity [14]. Oilseed flax following wheat and potato residues developed more tillers, branches, capsules, and higher test weights than continuous flax, with significant grain yield improvements in both rotation systems [15]. Wheat following faba bean in northern Ethiopia exhibited superior tiller density, grains per spike, and kernel mass compared to rotations with field pea, dekeko, or lentil [10]. Beyond its critical function in yield modulation, crop rotation significantly influences the formation of crop quality parameters. The spring wheat–winter rape–*Vicia sativa* rotation system outperformed wheat monocropping by elevating spike density, kernel number, thousand-kernel weight, and final yield, along with significant improvements in wheat grain protein concentration, sedimentation value, and wet gluten percentage [16]. The rice–morel and rice–milk vetch rotations enhanced milling recovery, visual characteristics, cooking/edible attributes, and nutritional components [17].

The variations in yield and quality across rotations stem from different influences of preceding crops on soil properties [18]. Rotating rubber dandelion with sugar beet elevates soil organic carbon (SOC), nitrogen (N), and plant-essential nutrients relative to the monoculture, thereby optimizing photosynthetic carbon partitioning and growth processes [19,20]. In sandy loam of the Tibetan Plateau, barley–wheat and barley–rapeseed rotations boosted yield by augmenting soil microbe N and C while reducing pH [21]. Under Loess Plateau conditions (loessial soil), oat-based rotations sustained elevated levels of crude protein, fat, ash, calcium, and phosphorus (P) in oat hay by enhancing soil health [22]. Research across Illinois in the USA demonstrated that short-duration corn rotations did not enhance soil health relative to continuous corn, irrespective of site-specific edaphic conditions [23]. For loamy soil in the North China Plain, legume-based rotations boosted topsoil ecological multifunctionality and preserved SOC sequestration relative to the corn–wheat system, consequently improving following wheat productivity [24]. Within drained Brookston clay loam in Canada, multi-year rotations enhanced productivity relative to sole wheat but exhibited deteriorating soil health parameters following soybean integration despite yield benefit [25].

With intensifying worldwide food security concerns, ensuring consistent grain provision and nutritional improvement is critically imperative, and soil properties profoundly determine agricultural productivity [26]. Thus, holistic research integrating yield, quality parameters, and pedological factors for wheat is fundamentally necessary. However, knowledge gaps persist regarding how covariation patterns and cause–effect relationships interconnect winter wheat productivity, grain protein attributes, and edaphic chemistry in saline–sodic marginal ecosystems with varied pre-crops. We examined five canonical rotations to (1) quantify pre-crop driven divergence in winter wheat yield, protein composition, and soil nutrients; (2) quantify synergistic interactions and coupled dynamics linking yield, grain quality, and soil nutrients; and (3) identify precursor crops optimally balancing yield-quality synergies with soil amelioration. This study would provide scientific foundations for refining local cropping systems.

## 2. Results

### 2.1. Effects of Preceding Crops on Yield and Yield Components in Winter Wheat 

**Figure 1:**
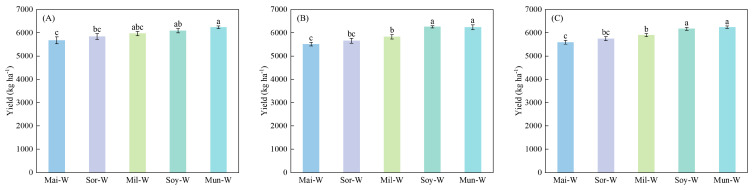
Impacts of preceding crops on the yield of subsequent winter wheat in 2022 (**A**), 2023 (**B**), and the average of 2022 and 2023 (**C**). Values are presented as means ± SE, with three replicates in 2022 and 2023 individually and six replicates for the combined data in 2022–2023. Different lowercases indicate significant differences between different rotation systems, analyzed by one-way ANOVA followed by the LSD test (*p* < 0.05).

Significant differences in wheat yield response to preceding crops were observed across different years (Figure 1). In 2022, wheat yield in Mun-W was 9.95% higher than in Mai-W. In 2023, Soy-W demonstrated a 13.57% yield advantage over the Mai-W. Over the 2022–2023 period, the Mun-W exhibited significantly higher grain yield compared to the Mai-W, representing an 11.56% advantage.

In 2022, leguminous precursors significantly increased tillers per plant and spike number per unit area of winter wheat compared to the gramineous crops (Table 1, *p* < 0.05). For effective tillers per plant, the top-performing Mun-W had 9.65% more than the lowest-performing Mil-W. In terms of spike number per unit area, the Mun-W was 4.23% higher than the Sor-W. The Mun-W exceeded a 14.91% kernel number per spike relative to the Mai-W. However, Soy-W showed a 6.53% lower 1000-kernel weight than Sor-W. In 2023, no significant differences in effective tillers per plant were observed among treatments (*p* > 0.05). TheSoy-W produced an advantage of 4.45% spike number per unit area and 14.62% kernel number per spike, but resulted in a diminution of 5.29%, compared to the Mai-W. Averaged over 2022–2023, the winter wheat in Mun-W showed the greatest effective tiller number per plant, spike number, and kernel number per spike among five rotations, but did not represent the 1000-kernel weight. There was a 6.28% difference in 1000-kernel weight between the Soy-W and Sor-W.

### 2.2. Effects of Preceding Crops on Protein Fractions in Winter Wheat 

**Figure 2:**
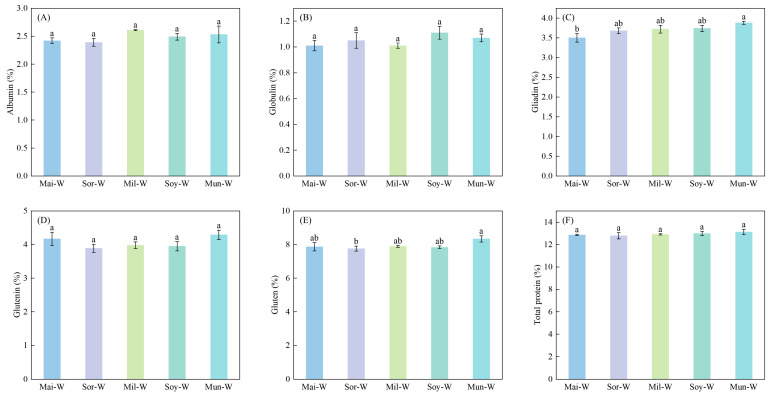
Impacts of different preceding crops on albumin (**A**), globulin (**B**), gliadin (**C**), glutenin (**D**), gluten (**E**), and total protein (**F**) of subsequent winter wheat in 2022. Values are presented as means ± SE, with three replicates in 2022. Different lowercases indicate significant differences between different rotation systems, analyzed by one-way ANOVA followed by the LSD test (*p* < 0.05).

**Figure 3:**
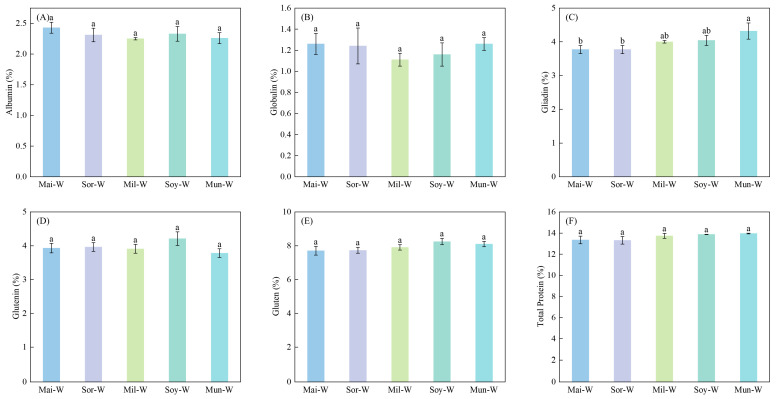
Impacts of different preceding crops on albumin (**A**), globulin (**B**), gliadin (**C**), glutenin (**D**), gluten (**E**), and total protein (**F**) of subsequent winter wheat in 2023. Values are presented as means ± SE, with three replicates in 2023. Different lowercases indicate significant differences between different rotation systems, analyzed by one-way ANOVA followed by the LSD test (*p* < 0.05).

**Figure 4:**
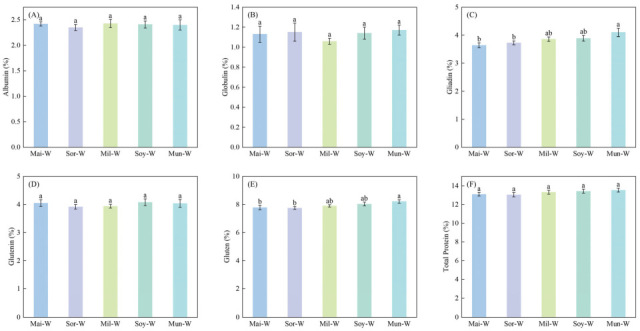
Impacts of different preceding crops on albumin (**A**), globulin (**B**), gliadin (**C**), glutenin (**D**), gluten (**E**), and total protein (**F**) of subsequent winter wheat from 2022 to 2023. Values are presented as means ± SE, with six replicates for the combined data in 2022–2023. Different lowercases indicate significant differences between different rotation systems, analyzed by one-way ANOVA followed by the LSD test (*p* < 0.05).

Preceding crop types markedly altered grain gliadin and gluten protein concentrations of following winter wheat in 2022 (Figure 2) and 2023 (Figure 3). Gliadin and gluten protein concentrations of winter wheat in Mun-W were, respectively, 10.86% and 7.47% higher than those of Mai-W and Sor-W in 2022. The Mun-W also performed the greatest gliadin content among five rotations in 2023. As indicated by 2022–2023 averaged data, the Mun-W was 12.64% higher in gliadin than the Mai-W and 6.06% higher in gluten than the Sor-W (Figure 4).

### 2.3. Effects of Preceding Crops on Soil Chemical Properties

During the initial rotation year (2022), soil-available N varied significantly across treatments (*p* < 0.05), yet preceding crops showed negligible impacts on other soil indicators (Table 2). Depressed soil AN was the key determinant for the minimal SFI of the Mai-W among all rotations. The Mun-W system, characterized by comprehensively elevated soil nutrients, demonstrated the highest SFI across treatments. The preceding crop effects on SOM, AN, AP, and AK were fully manifested following two complete rotation cycles by 2023. The highest SOM occurred in Mun-W rotation, which was significantly superior to Sor-W and Mai-W (*p* < 0.05), with its SOM being 0.66% higher than Sor-W and 1.32% higher than Mai-W. The Soy-W rotation achieved the maximum AP among treatments. The Mai-W maintained the lowest AP and AK. The SFI in Mun-W was 34.62% higher than Sor-W and 112.12% higher than Mai-W. Synthesizing cumulative legacy effects across both growing seasons (2022–2023), contrasting rotation regimes exhibited significant heterogeneity in soil pH, SOM, and SFI (*p* < 0.05). The pH in Mai-W was 0.36% higher than that in Soy-W. The Mun-W attained peak SOM and SFI, while the Mai-W had the lowest. Specifically, SOM in Mun-W was 0.99% higher than that in Mai-W, and SFI in Mun-W was 106.7% higher than that in Mai-W.

### 2.4. Relationship of Winter Wheat Yield, Grain Protein Quality, and Soil Chemical Properties

**Figure 5:**
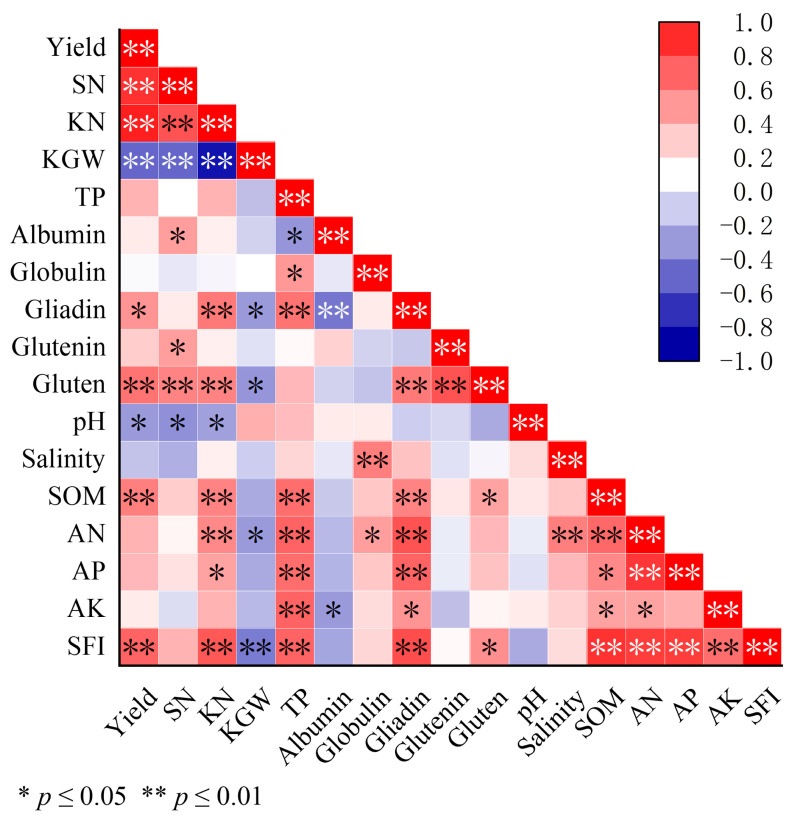
Relationship of winter wheat yield, grain protein quality, and soil chemical properties from 2022 to 2023. The dataset comprises all six replicate measurements from both 2022 and 2023. The correlation analyses are quantified via the Pearson correlation coefficient, with statistical significance evaluated at *p* < 0.05.

Across crop rotation regimes, winter wheat manifested negligible nitrogen dilution responses (Figure 5). However, grain yield and kernel number per spike (KN) demonstrated significant positive correlations with the gliadin and gluten protein content of grain (*p* < 0.05). Spike number per unit area (SN) of winter wheat displayed positive linkages with albumin, glutenin, and gluten in grain, whereas 1000-kernel weight (KGW) showed an inverse relationship (*p* < 0.05). Wheat yield demonstrated marked interrelations with soil pH, SOM, and SFI. The KN of winter wheat was most substantially influenced by soil chemical properties. The SN of winter wheat proved highly sensitive to soil pH variation, and kernel weight (KGW) was principally modulated by the AN and SFI (*p* < 0.05). Total grain protein and gliadin content showed significant positive correlations with soil salinity, SOM, AN, AP, AK, and SFI. Gluten protein likewise exhibited positive associations with SOM and SFI values.

### 2.5. Comprehensive Outcomes of Preceding Crop Types

**Figure 6:**
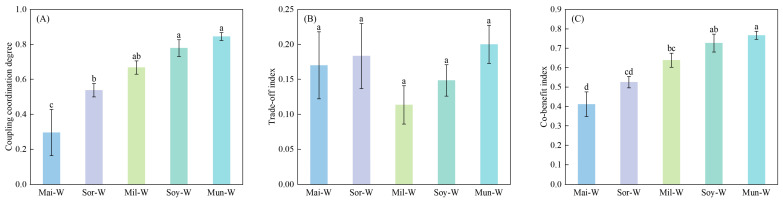
Comprehensive outcomes of preceding crop types across wheat yield, grain gluten, and soil fertility index. (**A**–**C**) indicated the coupling coordination degree, trade-off, and benefit index, respectively. Values are presented as means ± SE, with six replicates for the combined data in 2022–2023. Different lowercases indicate significant differences between different rotation systems, analyzed by one-way ANOVA followed by the LSD test (*p* < 0.05).

The preceding crop type exerted pronounced modulation of the CCD between grain yield, gluten accumulation, and soil fertility metrics (Figure 6A, *p* < 0.05). Legume–winter wheat rotations exhibited markedly superior CCD relative to cereal–winter wheat counterparts (*p* < 0.05). The CCD in legume–winter wheat systems exhibited significant superiority by 62.41% over the gramineous crop–winter wheat rotations. The Mun-W significantly outperformed other rotations, attaining maximum CCD, which was 180% higher than that in Mai-W.

The trade-off index (TOI) among the wheat yield, wheat grain gluten, and SFI showed no statistical divergence across preceding crop types (Figure 6B, *p* > 0.05). Co-benefit index (CBI) harmonizing wheat yield, grain gluten, and SFI were markedly elevated in legume–winter wheat rotations compared to gramineous crop–winter wheat systems (Figure 6C, *p* < 0.05). The CBI in legume–winter wheat systems exhibited significant superiority by 42.22% over the gramineous crop–winter wheat rotations. TheMun-W significantly achieved the maximum CBI among all rotations, which was 87.80% higher than that in Mai-W.

## 3. Discussion

### 3.1. Yield Variation in Winter Wheat Induced by Contrasting Pre-Crop Legacies

This study revealed that across individual years (2022, 2023) and their biennial mean, leguminous crops (mung bean and soybean) as preceding crops consistently produced a higher positive effect on the grain yield of winter wheat. The mung bean–millet and soybean–maize rotations increased subsequent yield of millet [27] and maize [28]. This pre-crop legacy effect proved particularly pronounced in marginal saline-alkali soil with inferior soil quality, with legume precursors boosting subsequent wheat yield by 51.3% to 54.6% [29]. Winter wheat in legume-based rotations achieved yield augmentation via improvement in spike number per hectare and kernel number per spike in this study. Specifically, the Mun-W system enhanced 4.05% greater spike number per hectare and 14.31% higher kernel number per spike than the Mai-W system. Contrastingly, Ingver et al. suggested legumes as preceding crops may enhance 1000-kernel weight in following winter wheat [30]. This discrepancy may be ascribed to the N fertilization rate difference between two studies, where adding N promoted wheat tillering, and a high spike number further reduced the grain filling efficiency and KGW [31]. Legume–cereal rotations exhibit greater yield advantages over cereal–cereal systems due to nutrient complementary demands [24] and enhanced soil nitrogen availability from legumes through biological N_2_ fixation and high-quality rhizodeposition [11]. Our study showed maize as the lowest-yielding precursor for winter wheat. The Mun-W produced 11.56% higher yield than the Mai-W across 2022–2023. These differences may be due to nutrient demand overlap in gramineous sequences causing progressive soil depletion, exacerbated by maize’s high N demand creating a post-harvest N deficit for wheat. The higher soil AN after two rotation cycles from 2022 to 2023 confirmed this.

### 3.2. Effects of Preceding Crop Types on Protein Quality in Winter Wheat

Wheat protein constitutes a vital nutritional source in human diets, and protein concentration and fraction composition in wheat kernels directly determine end-product processing quality and nutritional implications for human health. The leguminous crop significantly increased the gliadin and gluten protein content of subsequent winter wheat, especially for the mung bean. In 2022, the Mun-W system had 10.86% higher gliadin and 7.47% higher gluten protein than the Mai-W system. In 2023, the gliadin in Mun-W was 14.59% higher than that in the Mai-W and Sor-W. This is consistent with previous research finding that leguminous crops enhanced gluten protein content in subsequent wheat crops [32]. This phenomenon arises as leguminous precursors boost total soil N and available N via biological nitrogen fixation, improving nitrogen assimilation efficiency in the following crop and ultimately providing sufficient N substrates for winter wheat gluten production [33]. Additionally, legumes enhance soil fertility through residual effects, which improved N use efficiency in subsequent winter wheat, consequently promoting superior grain protein composition [34]. The high N content and low C:N ratio of legume root nodule deposits stimulated microbial activity and accelerated N mineralization and available N release, providing abundant nitrogen for subsequent winter wheat crops [35,36]. Decomposition dynamics and C-N release characteristics of leguminous green manure in the farmland of the Lhasa River Valley corroborated the persistent supply of soil-available N following legume rotation, fulfilling N demand for gluten protein synthesis during wheat grain filling [37].

However, when sorghum or maize served as the preceding crop, gluten protein content in subsequent winter wheat was significantly reduced compared to the legume–winter wheat rotations. The high C/N ratio in gramineous rhizodeposition may induce stronger microbial nitrogen immobilization, slower turnover, and reduced nutrient availability [35], and N derived from rhizodeposition from maize exhibited lower quantity and poorer proportional allocation within soil macroaggregates compared to the soybean [38]. Consequently, soil-available N reserves in Mai-W and Sor-W were lower than that in Soy-W following cereal crop harvesting. This directly caused inadequate N supply for protein synthesis during late grain development in winter wheat [39], diminishing grain nitrogen accumulation and gluten protein level. Additionally, the high C/N ratio of gramineous residues in soil elevated recalcitrant components (such as cellulose and lignin) and slowed the N mineralization, which constrained nitrogen supply for winter wheat gluten protein synthesis [40].

### 3.3. Soil Fertility Response to Rotations

Soil chemical attributes constitute essential metrics for evaluating soil fertility [41], and the soil fertility index operates as a composite quantifier synthesizing diverse soil indicators routinely employed in soil fertility assessment. The SFI proves crucial for addressing field heterogeneity and optimizing crop productivity with minimal environmental footprint [42]. A well-designed crop rotation significantly enhances soil fertility and upgrades the yield and quality of subsequent crops [27,43]. We examined the effects of five different pre-crops on soil chemical characteristics in a winter wheat system. The Mun-W rotation achieved the peak SFI, exceeding that in Sor-W by 34.62% and Mai-W by 112.12%. In the initial rotation year, it solely exhibited significant effects on soil AN. Upon reaching the second rotation cycle, legume-based winter wheat rotations showed markedly elevated levels of SOM, AN, AP, and AK relative to the maize–winter wheat rotation, driven by the cumulative legacy effect of preceding crops [44]. This phenomenon could be attributed to rhizobial symbiotic nitrogen fixation in legumes, which converts atmospheric nitrogen into plant-available form and increases available nitrogen reserves [45]. Legumes exude greater quantities of organic acid and protons from their root system, reducing soil pH and increasing the soil-available phosphorus concentration. The superior capacity of legumes to augment SOM and generate organic acid in its decomposition activates the inherent soil phosphorus reserves, augmenting soil-available phosphorus concentration [46,47]. The greatest soil-available potassium was observed in the Mil-W rotation. Species-unique rhizodeposits of miller potentially modulate rhizospheric microbiomes and soil physicochemical characteristics, facilitating potassium release in the soil [48]. The SOM was higher when legumes served as the preceding crop, with the highest SOM content observed following mung beans. The Mun-W showed an advantage of 0.99% in SOM compared to the Mai-W, which aligns with previous research findings [49,50]. The improved topsoil structure and increase in SOC and N in the aggregate potentially enhanced the storage of SOC in crop rotations [51,52,53]. This may be because enhanced nitrogen input from biological nitrogen fixation by leguminous pre-crops could stimulate crop growth and soil organic carbon residue [51,54].

### 3.4. Holistic Performance in Diverse Preceding Crops—Winter Wheat Rotation

Mung bean–winter wheat achieved a higher coupling coordination degree among winter wheat yield, gluten protein content, and soil fertility index, significantly exceeding all three gramineous predecessor-based rotations (maize, sorghum, and millet). The CCD in legume–winter wheat systems exhibited significant superiority by 62.41% over the gramineous crop–winter wheat rotations. However, the trade-off index showed no significant differences across preceding crop types, and significant differences existed in the co-benefit index. This indicates that leguminous preceding crops facilitate synchronous optimization of winter wheat yield, protein quality enhancement, and soil fertility improvement, especially for mung bean. The CBI in legume–winter wheat systems manifested significant advantage of 42.22% over the gramineous crop–winter wheat rotations, and the Mun-W significantly exceeded other rotations, reaching the maximum co-benefit index of 0.77. In contrast, gramineous predecessors, particularly maize and sorghum, prove less effective in achieving tripartite synergistic benefits.

Probing the synergistic interplay and trade-off among the three factors, coupled with correlational analysis, revealed that soil pH and SOM serve as critical mediators governing how pre-crops affect subsequent winter wheat yield and grain quality. Leguminous predecessors achieved the greatest legacy effect on winter wheat yield. This was attributed to the fact that legumes provide more SOM and enhance soil enzyme activities and microbial biomass, ultimately evaluating the nutrient bioavailability for plant uptake [55]. Counteractively, elevated soil pH coupled with reduced organic matter content were key determinants of its lowest yield performance in maize–wheat rotation. Our data align with Sharma et al. on high pH-induced yield loss [56]. Leguminous crops increase the mean mineralization rates of both N and C, thereby strengthening the stabilizing feedback between soil nitrogen availability and biological nitrogen fixation [57]. This process augments total SOC and N pools, providing more substrates for winter wheat gluten protein synthesis [57,58,59]. Leguminous preceding crops conferred dual advantages in winter wheat yield and gluten protein, which is consistent with prior research findings. Soybean increased both gluten protein content and yield in subsequent wheat compared to oilseed [32], which is mainly associated with soil enrichment with plant-AN following soybean cultivation. Therefore, when legumes serve as the preceding crop, they optimize SOM, AN, and pH, achieving the highest synergy performance among winter wheat yield, grain protein quality, and soil chemical properties.

## 4. Materials and Methods

### 4.1. Site Description

Huanghua City is located at 38°09′–38°39′ N, 117°05′–117°49′ E. This region comprises vast coastal alluvial deposits forming subdued, flat plains that gently dip northeastward with altitudes ranging from 1 to 5 m. Mean annual wind speed is 4.2 m/s, mean sunshine duration 2755 h, mean frost-free period 194 days, mean temperature 12.1 °C, and mean precipitation 627 mm (75% in summer) [60], resulting in frequent spring droughts, summer floods, and autumn moisture deficits. Potable groundwater resides 320–600 m below the surface with total dissolved solids of 1.1 g/kg and SOM of 14.8 g/kg. The region suffers severe freshwater scarcity with a brackish nature, heterogeneous availability, and excessive fluoride levels. Severe land salinization occurs ubiquitously across the region, and the soil type primarily is fluvo-aquic soil that is characterized by a clayey texture and slight salinization.

### 4.2. Experimental Design

The experiment comprised five treatments: maize–winter wheat (Mai-W), millet–winter wheat (Mil-W), sorghum–winter wheat (Sor-W), soybean–winter wheat (Soy-W), and mung bean–winter wheat (Mun-W) rotations. Each treatment was replicated three times. Individual plot dimensions measured 10 m × 4.8 m (48 m^2^). The winter wheat cultivar Jiemai-19 was cultivated in this trial, whose suitable cultivation areas cover dry, barren saline-alkaline land in the Hebei, Tianjin, and Shandong regions of China. Field evaluations confirm a drought resistance index of 1.2 under natural aridity stress. It exhibits resilience against moderate drought events and possesses advantages including high temperature tolerance, strong cold resistance and lodging resistance, and favorable maturation under low-light conditions. This cultivar was sown at 20 cm row spacing, with 10 kg ha^−1^. Sowing dates were 11 October 2021 and 15 October 2022, respectively, and wheat was harvested on 7 June 2022 and 9 June 2023 for the respective growing seasons. Pre-sowing basal application of N-P-K compound fertilizer (20-15-10) was carried out at 500 kg ha^−1^, followed by 90 kg N ha^−1^ (urea) supplementation at the jointing phase of wheat. Chemical interventions, including herbicides, were excluded throughout the experiment. Excepting fertilizer applications, identical agronomic practices were maintained for all plots.

### 4.3. Measurement of Soil Chemical Properties

After the winter wheat harvest, soil samples from the 0–20 cm depth were collected using the two-point sampling method, conducted in the row and interrow of the wheat plot. For each plot, triplicate samples were obtained to guarantee analytical robustness. Collected soil samples underwent shade-air-drying pretreatment and passing through a 2 mm sieve before measuring all relevant properties. Soil samples were then analyzed for pH, salinity, organic matter, alkali-hydrolyzed nitrogen, available phosphorus, and available potassium. SOM was determined using the potassium dichromate-concentrated sulfuric acid external heating method. pH was measured using the potentiometric method. Soluble salt content in the soil was determined using the gravimetric method. Alkali-hydrolyzed nitrogen (AN) content in the soil was determined using the diffusion method. Available P (AP) and available potassium (AK) were determined using the NaHCO_3_ extraction-Mo-Sb colorimetric spectrophotometric method and the NH_4_OAc extraction-flame photometric method, respectively. Standard procedures for soil parameter quantification were implemented in accordance with established methodologies [61]. Finally, all soil chemical indicators across the five treatments were normalized. The soil fertility index (SFI) for the five treatments was calculated using the entropy weight method [62]. The specific steps are as follows:

Normalization was performed for all soil chemical properties (pH, salinity, SOM, AN, AP, and AK). Positive indicators were processed according to Equation (1); negative indicators were processed according to Equation (2):

(1) $$x_{ij}^{\prime }=\frac{x_{ij}-min\left(x_{j}\right)}{max\left(x_{j}\right)-min\left(x_{j}\right)}$$

(2) $$x_{ij}^{\prime }=\frac{max(x_{j})-x_{ij}}{max(x_{j})-min(x_{j})}$$

In Equations (1) and (2): $x_{ij}^{\prime }$ is the normalized value of the i-th observation for the j-th chemical indicator; $x_{ij}$ is the selected quantity of the i-th observation for the j-th chemical indicator; $min(x_{j})$ and $max(x_{j})$ are the minimum value and maximum value of the j-th chemical indicator, respectively.

The proportion $y_{ij}$ of the i-th observation for the j-th chemical indicator is:

(3) $$\begin{array}{ccc} y_{ij} & = & \frac{x_{ij}^{\prime }}{\sum_{i=1}^{m}\;x_{ij}^{\prime }} \end{array}$$

In Equation (3): m is the number of chemical indicators.

The information entropy of the j-th chemical indicator is:

(4) $$e_{j}=-K\sum_{i=1}^{m}\;y_{ij}\ln y_{ij}$$

(5) $$K=\frac{1}{\ln m}$$

In Equations (4) and (5): K = 1/ln m, K is a constant.

The weight of the j-th chemical indicator is:

(6) $$w_{j}=\frac{1-e_{j}}{\sum_{k=1}^{n}\;\left(1-e_{k}\right)}$$

In Equation (6), *n* is the number of observations for each chemical indicator.

The comprehensive score (soil fertility index ${SFI}_{i}$) for the i-th observation was obtained through weighted calculation:

(7) $${SFI}_{i}=\sum_{j=1}^{n}\;y_{ij}w_{j}$$

Subsequently, the soil fertility index was calculated for each treatment.

### 4.4. Measurement of Yield and Its Components in Winter Wheat

At wheat maturity, five spike samples (each from a 2 m row segment) were randomly collected per plot to determine spike number per unit area (SN) and grain yield. Twenty spikes per sample were used for determining kernel number per spike (KN). Twenty spikes from each sample were randomly selected to measure kernel number per spike. Five duplicates were selected per plot for 1000-kernel weight (KGW) measurement, following threshing of collected spikes.

### 4.5. Measurement of Protein Composition in Winter Wheat Grain

The sequential extraction method was used to extract albumin, globulin, gliadin, and glutenin contents from wheat grains [63].

### 4.6. Trade-Off and Co-Benefit Indices

The entropy weight method was applied to normalize and calculate weights for three indicators: yield, gluten protein, and soil fertility index, following the same procedures as (1)–(6). Subsequently, trade-off and co-benefit indices were systematically calculated between each pair of indicators [64].

(8) $$TOI=\sqrt{\frac{1}{n-1}\sum_{i=1}^{n}\;(S_{i}-\bar{S}{)}^{2}}$$

In the formula, TOI is the trade-off index of *n* indicators. $S_{i}$ is the normalized value of the ith indicator by the Min-Max scaling method. $\bar{S}$ is the average value of *n* indicators.

(9) $$CBI=\sqrt{\sum_{i=1}^{n}\;w_{i}S_{i}\left(1-TOI\right)}$$

In the formula, CBI is the co-benefit index of *n* indicators, $w_{i}$ is the weight of the ith indicator, and *n* is the number of indicators.

### 4.7. Coupling Coordination Degree

The entropy weight method was applied to normalize and calculate weights for three indicators: winter wheat yield, gluten protein content, and integrated soil fertility index, following the same procedures as (1)–(6). Subsequently, the coupling coordination degree (CCD) was systematically calculated between each pair of indicators [65].

(10) $$C_{n}=n\times {\left[\frac{u_{1}u_{2}\ldots u_{n}}{{\left(u_{1}+u_{2}+\ldots +u_{n}\right)}^{n}}\right]}^{\frac{1}{n}}$$

In Equation (10), *C* is the coupling degree of *n* indicators and $u_{n}$ is the comprehensive score of each indicator.

(11) $$T=\alpha_{1}u_{1}+\alpha_{2}u_{2}+\ldots +\alpha_{n}u_{n}$$

In Equation (11), T is the coordination degree of *n* indicators and *α_n_* is the weight of u*_n_*.

(12) $$CCD=\sqrt{C\times T}$$

In Equation (12), CCD is the coupling coordination degree index.

### 4.8. Statistical Analysis

One-way ANOVA was used to assess differences in the grain yield, effective tiller number per plant, yield components (spike number per hectare, kernel number per spike, 1000-kernel weight), protein compositions (total N, albumin, globulin, gliadin, and glutenin proteins), soil chemical properties (pH, salinity, SOM, AN, AP, AK, SFI), CCD, TOI, and CBI, using the least significant difference (LSD test at a significance level of *p* < 0.05 (SPSS 25.0, IBM, Armonk, NY, USA). The correlation analyses among the above measurements and calculated values are quantified by the Pearson correlation coefficient at a significance level of *p* < 0.05. All data presented in the figures and tables are expressed as the mean ± standard error (SE). The figures were generated with Origin 2024 (OriginLab Corp., Northampton, MA, USA).

## 5. Conclusions

Benefiting from increased spike and kernel number per unit, leguminous predecessors substantially boost winter wheat yield without triggering the nitrogen dilution effect. Winter wheat in the Mun-W rotation increased 11.56% of yield and even elevated 5.61% of grain gluten protein compared to that in Mai-W. Following two complete rotation cycles, pre-crop legacy impacts on soil were markedly amplified, resulting in significantly higher levels of SOM, AN, AP, AK, and SFI in legume–winter wheat rotations compared to the maize–winter wheat system. Governed by the tripartite correlations between winter wheat yield, grain protein quality, and soil chemical properties, the rotation initiated with legumes optimized the coupling coordination degree and co-benefit index integrating grain yield, gluten content, and soil fertility, and suppressed the trade-off effect among these parameters. Legume–winter wheat systems demonstrated significantly higher CCD (62.41%) and CBI (42.22%) relative to gramineous crop–winter wheat rotations. Notably, the Mun-W achieved 180.0% higher CCD and 87.80% greater CBI than the Mai-W. Consequently, legumes are more suitable as the preceding crop for winter wheat than the gramineous crop; particularly, the mung bean–winter wheat rotation demonstrated a synergy benefit of 0.77. Critically, the protein quality metric in this synergistic effect solely employed gluten protein as the responsive indicator, and soil fertility assessment was confined to key chemical properties. This limitation resulted in the holistic soil properties and crop quality being insufficient to characterize. These constraints compel forthcoming studies examining the synergy effect of the rotation on crop and soil to broaden observational parameters and implement the long-term positioning experiment.

## Figures and Tables

**Table 1:** The impact of different preceding crops on yield components of winter wheat in 2022–2023.

Year	Preceding Crop Types	Effective Tiller Number per Plant	Spike Number (10^4^ ha^−1^)	Kernel Number per Spike	1000-Kernel Weight (g)
2022	Mai-W	1.16 ± 0.03 b	513.10 ± 3.52 b	27.50 ± 0.40 c	47.30 ± 0.26 a
	Sor-W	1.15 ± 0.02 b	512.60 ± 6.36 b	28.20 ± 0.15 c	47.50 ± 0.25 a
	Mil-W	1.14 ± 0.02 b	516.40 ± 2.06 b	30.40 ± 0.42 b	44.70 ± 0.21 b
	Soy-W	1.22 ± 0.03 a	530.80 ± 3.97 a	31.20 ± 0.44 ab	44.40 ± 0.12 b
	Mun-W	1.25 ± 0.02 a	534.30 ± 2.30 a	31.60 ± 0.19 a	44.50 ± 0.12 b
2023	Mai-W	1.11 ± 0.04 a	501.30 ± 1.64 b	27.57 ± 0.12 c	47.20 ± 0.20 a
	Sor-W	1.15 ± 0.04 a	502.40 ± 4.73 b	28.13 ± 0.19 c	47.30 ± 0.31 a
	Mil-W	1.18 ± 0.05 a	501.70 ± 4.41 b	30.37 ± 0.35 b	45.10 ± 0.30 b
	Soy-W	1.20 ± 0.03 a	523.60 ± 3.46 a	31.60 ± 0.12 a	44.80 ± 0.35 b
	Mun-W	1.17 ± 0.03 a	521.20 ± 0.12 a	31.27 ± 0.15 a	45.00 ± 0.62 b
2022–2023	Mai-W	1.13 ± 0.06 b	507.20 ± 7.74 b	27.53 ± 0.46 d	47.25 ± 0.37 a
	Sor-W	1.15 ± 0.05 ab	507.00 ± 10.32 b	28.17 ± 0.27 c	47.40 ± 0.45 a
	Mil-W	1.16 ± 0.06 ab	509.05 ± 9.66 b	30.38 ± 0.59 b	44.90 ± 0.46 b
	Soy-W	1.21 ± 0.05 a	527.20 ± 6.99 a	31.40 ± 0.54 a	44.60 ± 0.46 b
	Mun-W	1.21 ± 0.06 a	527.75 ± 7.61 a	31.47 ± 0.34 a	44.75 ± 0.75 b

Note: Values are presented as means ± SE, with three replicates in 2022 and 2023 individually and six replicates for the combined data in 2022–2023. Different lowercases indicate significant differences between different rotation systems, analyzed by one-way ANOVA followed by the LSD test (*p* < 0.05).

**Table 2:** Impacts of different crops–winter wheat rotation systems on topsoil chemical properties.

Year	Preceding Crop Types	pH	Salinity (g kg^−1^)	SOM (g kg^−1^)	AN (mg kg^−1^)	AP (mg kg^−1^)	AK (mg kg^−1^)	SFI
2022	Mai-W	8.32 ± 0.01 a	1.07 ± 0.01 a	15.11 ± 0.02 a	60.83 ± 1.08 b	11.57 ± 0.1 a	173.23 ± 1.48 a	0.27 ± 0.03 c
	Sor-W	8.3 ± 0.02 a	1.06 ± 0.02 a	15.11 ± 0.02 a	61.65 ± 0.45 b	11.72 ± 0.03 a	173.87 ± 1.71 a	0.36 ± 0.08 bc
	Mil-W	8.30 ± 0.01 a	1.07 ± 0.00 a	15.15 ± 0.06 a	63.96 ± 0.14 a	11.73 ± 0.02 a	173.96 ± 0.23 a	0.42 ± 0.05 abc
	Soy-W	8.29 ± 0.01 a	1.07 ± 0.01 a	15.14 ± 0.05 a	65.08 ± 0.58 a	11.93 ± 0.35 a	175.07 ± 1.72 a	0.48 ± 0.05 ab
	Mun-W	8.29 ± 0.01 a	1.08 ± 0.02 a	15.2 ± 0.02 a	64.61 ± 0.53 a	11.92 ± 0.31 a	175.61 ± 0.83 a	0.53 ± 0.03 a
2023	Mai-W	8.33 ± 0.02 a	1.11 ± 0.01 a	15.11 ± 0.02 c	66.35 ± 0.4 b	12.01 ± 0.08 b	174.10 ± 1.22 b	0.33 ± 0.05 c
	Sor-W	8.32 ± 0.02 a	1.10 ± 0.01 a	15.21 ± 0.03 b	66.22 ± 0.59 b	12.13 ± 0.10 ab	176.42 ± 0.82 ab	0.52 ± 0.07 b
	Mil-W	8.32 ± 0.00 a	1.10 ± 0.03 a	15.23 ± 0.03 ab	65.86 ± 0.35 b	12.15 ± 0.06 ab	178.07 ± 0.73 a	0.56 ± 0.05 ab
	Soy-W	8.30 ± 0.02 a	1.10 ± 0.02 a	15.23 ± 0.05 ab	67.86 ± 0.35 a	12.66 ± 0.14 a	175.95 ± 1.24 ab	0.64 ± 0.02 ab
	Mun-W	8.31 ± 0.02 a	1.10 ± 0.01 a	15.31 ± 0.02 a	67.89 ± 0.53 a	12.5 ± 0.33 ab	176.67 ± 0.88 ab	0.70 ± 0.06 a
Average	Mai-W	8.33 ± 0.02 a	1.09 ± 0.03 a	15.11 ± 0.03 b	63.59 ± 3.27 a	11.79 ± 0.28 a	173.66 ± 2.16 a	0.30 ± 0.03 c
	Sor-W	8.31 ± 0.03 ab	1.08 ± 0.03 a	15.16 ± 0.07 b	63.94 ± 2.64 a	11.93 ± 0.25 a	175.14 ± 2.50 a	0.44 ± 0.06 b
	Mil-W	8.31 ± 0.01 ab	1.09 ± 0.03 a	15.19 ± 0.08 ab	64.91 ± 1.12 a	11.94 ± 0.24 a	176.02 ± 2.41 a	0.49 ± 0.05 ab
	Soy-W	8.30 ± 0.03 b	1.09 ± 0.03 a	15.19 ± 0.09 ab	66.47 ± 1.69 a	12.29 ± 0.57 a	175.51 ± 2.38 a	0.56 ± 0.04 ab
	Mun-W	8.30 ± 0.02 ab	1.09 ± 0.02 a	15.26 ± 0.07 a	66.25 ± 1.97 a	12.21 ± 0.59 a	176.14 ± 1.45 a	0.62 ± 0.05 a

Note: SOM, soil organic matter; AN, alkali-hydrolyzed nitrogen; AP, available phosphorus; AK, available potassium; SFI, soil fertility index. Values are presented as means ± SE, with three replicates in 2022 and 2023 individually and six replicates for the combined data in 2022–2023. Different lowercases indicate significant differences between different rotation systems, analyzed by one-way ANOVA followed by the LSD test (*p* < 0.05).

## Data Availability

The data presented in this study are available upon request from the corresponding author.

## Abbreviations

The following abbreviations are used in this manuscript:

SOC	Soil organic carbon
N	Nitrogen
C	Carbon
P	Phosphorus
SOM	Soil organic matter
Mai-W	Maize–winter wheat rotation
Mil-W	Millet–winter wheat rotation
Sor-W	Sorghum–winter wheat rotation
Soy-W	Soybean–winter wheat rotation
Mun-W	Mung bean–winter wheat rotation
AN	Alkali-hydrolyzed nitrogen
AP	Available phosphorus
AK	Available potassium
SFI	Soil fertility index
SN	Spike number per unit
KN	Kernel number per spike
KGW	1000-kernel weight
TOI	Trade-off index
CBI	Co-benefit index
CCD	Coupling coordination degree
